# Skin Pigmentation Impacts the Clinical Diagnosis of Wound Infection: Imaging of Bacterial Burden to Overcome Diagnostic Limitations

**DOI:** 10.1007/s40615-023-01584-8

**Published:** 2023-04-11

**Authors:** Jonathan Johnson, Alton R. Johnson, Charles A. Andersen, Martha R. Kelso, Alisha R. Oropallo, Thomas E. Serena

**Affiliations:** 1 Comprehensive Wound Care Services and Capital Aesthetic & Laser Center, Washington, DC USA; 2 University of Michigan School of Medicine in the Division of Metabolism, Endocrinology and Diabetes-Podiatry, Ann Arbor, MI USA; 3 Madigan Army Medical Center Joint Base Lewis-McChord, Tacoma, WA USA; 4 Wound Care Plus LLC, Blue Springs, MO, USA; 5 Comprehensive Wound Healing Center and Hyperbarics, Northwell Health and Department of Vascular Surgery, Zucker School of Medicine Hofstra/Northwell, Hempstead, NY USA; 6 SerenaGroup Research Foundation, Cambridge, MA USA

**Keywords:** Bacterial load, Erythema, Fluorescence imaging, Infection, Medical education, Skin pigmentation

## Abstract

Underrepresentation of diverse skin tones in medical education and providers’ implicit racial bias drives inequities in wound care, such as disproportionally poor outcomes for Black patients. Diagnostic indicators (e.g., erythema) can present differently depending on skin pigmentation. This post hoc analysis of 350 chronic wounds from a prospective 14-site clinical trial aimed to determine how the perception of clinical signs and symptoms of infection (CSS) differs by patient skin tone and if fluorescence-imaging can offer a more objective diagnostic solution. Participants were grouped by skin tone (low, medium, high) as measured by the Fitzpatrick Skin Phototype Classification (FSPC) scale. CSS and total bacterial load (TBL) were compared across FSPC groups, along with sensitivity to detect TBL >10^4^ CFU/g using CSS alone and combined with fluorescence-imaging. Erythema was reported less often with increasing FSPC score (*p* = 0.05), from 13.4% (low), to 7.2% (medium), to 2.3% (high), despite comparable bacterial loads (median = 1.8 × 10^6^ CFU/g). CSS sensitivity in the high group (2.9%) was 4.8-fold to 8.4-fold lower than the low (*p* = 0.003) and medium groups (*p* = 0.04). Fluorescence-imaging significantly improved the detection of high bacterial load in each group, peaking in the high group at 12-fold over CSS alone. These findings underscore the threat of pervasive racialized health inequities in wound care, where missed diagnosis of pathogenic bacteria and infection could delay treatment, increasing the risk of complications and poor outcomes. Fluorescence-imaging is poised to fill this gap, at least in part, serving as a more objective and equitable indicator of wound bacteria. Clinicaltrials.gov#NCT03540004 registered 16-05-2018.

## Introduction

Omittance of diverse skin tones in medical textbooks and literature drives healthcare inequities in racial and ethnic minority patient populations [1]. A systematic review of diversity in dermatologic randomized control trials (RCTs) showed that, while racial and ethnic data reporting is becoming increasingly transparent, racially inclusive representation remains stagnant [2]. As medical specialties push towards dismantling structural racism [3], more evidence is needed to understand how skin tone influences the appearance of chronic wounds and how their misinterpretation can affect patient outcomes [4].

Studies of US Medicare beneficiaries show that patients who identify as Black and/or live in rural areas have up to a 10% increased risk of major leg amputation or death [5, 6]. Other US studies report that pressure ulcers/injuries (PUs/PIs) are more likely to form [7–9], are more severe [10], and are less likely to heal after 90 days [11] in Black nursing home residents than in White residents. The reason behind these disproportionally poor wound outcomes is multifactorial. Contributing factors include inequities in social determinants of health experienced by underprivileged populations [12], but also a failure of healthcare providers to recognize pathognomonic skin changes signaling wound development and progression. A prime example of this is erythema, which varies in appearance depending on skin tone [13]. Though PU/PI-specific guidelines are beginning to include education on detecting erythema in highly pigmented skin [14], similar guidance for other wound types and pathologies (e.g., infection) is minimal.

Recognizing wound infection and associated complications in their early stages is a priority; it allows for preventative measures and/or early interventions to circumvent devastating consequences on patient quality of life, morbidity, and mortality [15, 16]. However, despite numerous wound infection assessment tools [16], equitable assessment across skin tones remains a challenge. In a global survey of 226 health care providers, “red legs” (i.e., erythema) and wound infection were among the top pathologies listed as challenging to assess on dark skin tones [17]. Another barrier to equitable wound assessment is biased clinical terminology such as “redness.” Often an inaccurate descriptor of erythema in highly pigmented skin [4], this term is used synonymously in some wound infection assessment mnemonics [18, 19]. Other key diagnostic indicators in wound assessment (e.g., cyanosis, inflammation, discoloration, blanching, scarring) also vary considerably in presentation depending on skin pigmentation [20]. This challenge is compounded in chronic wound patients, where underlying comorbid conditions (e.g., diabetes) frequently alter or compromise immune function and expression of infection [16, 21]. Consequently, we speculate that clinical indicators of infection are more likely to be unrecognized or underappreciated when considering wounds on highly pigmented skin, potentially leading to delayed or missed diagnoses and worsened wound outcomes.

We analyzed clinical trial findings from 20 US clinicians and quantitative culture data from 350 chronic wounds to understand the occurrence and potential drivers of disparities in wound infection assessment across skin tones. We assessed the frequency of numerous clinical signs and symptoms (CSS) of wound infection and determined their sensitivity in detecting levels of bacteria usually considered pathologic (healing delays, infection). We also explored fluorescence imaging (FL-imaging) of wound bacteria, also used in this trial, as an objective and more equitable diagnostic.

## Methods

### Study Population and Design

This post hoc analysis evaluated data from 350 chronic wounds from the Fluorescence Imaging Assessment and Guidance (FLAAG) clinical trial, a prospective, single-blind, multicenter cross-sectional clinical trial (clinicaltrials.gov#NCT03540004) [22] of 350 adults (>18 years) presenting to 14 US outpatient advanced wound centers between May 2018 and April 2019. The wounds were of unknown infection status and were examined and imaged by 20 experienced wound care clinicians including surgeons, podiatrists, and nurse practitioners. Exclusion criteria consisted of treatment with an investigational drug within the last month, a recent wound tissue culture (<30 days), inability to consent, or an anatomical location that could not be imaged. The study was approved by an external Institutional Review Board (Veritas IRB, Montreal, QC).

### Assessment of Clinical Signs and Symptoms and Fluorescence Imaging of Wound Bioburden

Clinicians were trained prior to the study on how to perform and interpret FL-imaging (didactic and hands-on training; clinicians were required to pass an image interpretation certification test with a score of >80%). Clinicians reviewed each patient’s medical history, assessed their wound for all covert, overt, and spreading CSS (as indicated by the International Wound Infection Institute (IWII) 2016 guidelines [23]), and recorded any detected CSS (or their complete absence). Wounds were considered positive for CSS based on the detection of ≥3 IWII criteria. Immediately following clinical assessment, clinicians used a FL-imaging device (MolecuLight ***i:****X*, Toronto, Canada) to capture standard and fluorescence images of the wound. This noninvasive, contrast agent-free technology identifies regions of high (>10^4^ CFU/g) bacterial burden in and around wounds [22, 24], where red fluorescence signals the presence of endogenously produced porphyrins from bacteria [25] and cyan fluorescence is indicative of pyoverdine from *Pseudomonas aeruginosa* [26]. A high positive predictive value (>93%) [26, 27] has been validated for red and cyan fluorescence signals corresponding to the presence and location of most bacterial species [25] at loads >10^4^ CFU/g.

### Quantitation of Total Bacterial Load in Wounds

Tissue samples were obtained from each wound after cleansing for debris and/or surface contamination, as previously described [22, 28]. Tissue samples were analyzed by a CLIA-certified laboratory (Eurofins Central Laboratory, Lancaster, PA) for bacterial load and species using aseptic, gold-standard techniques. Quantitative culture was performed as previously described [22], where microbiologists were blinded to the results of CSS assessment and FL-imaging.

### Fitzpatrick Skin Type Classification

**Fig. 1:**
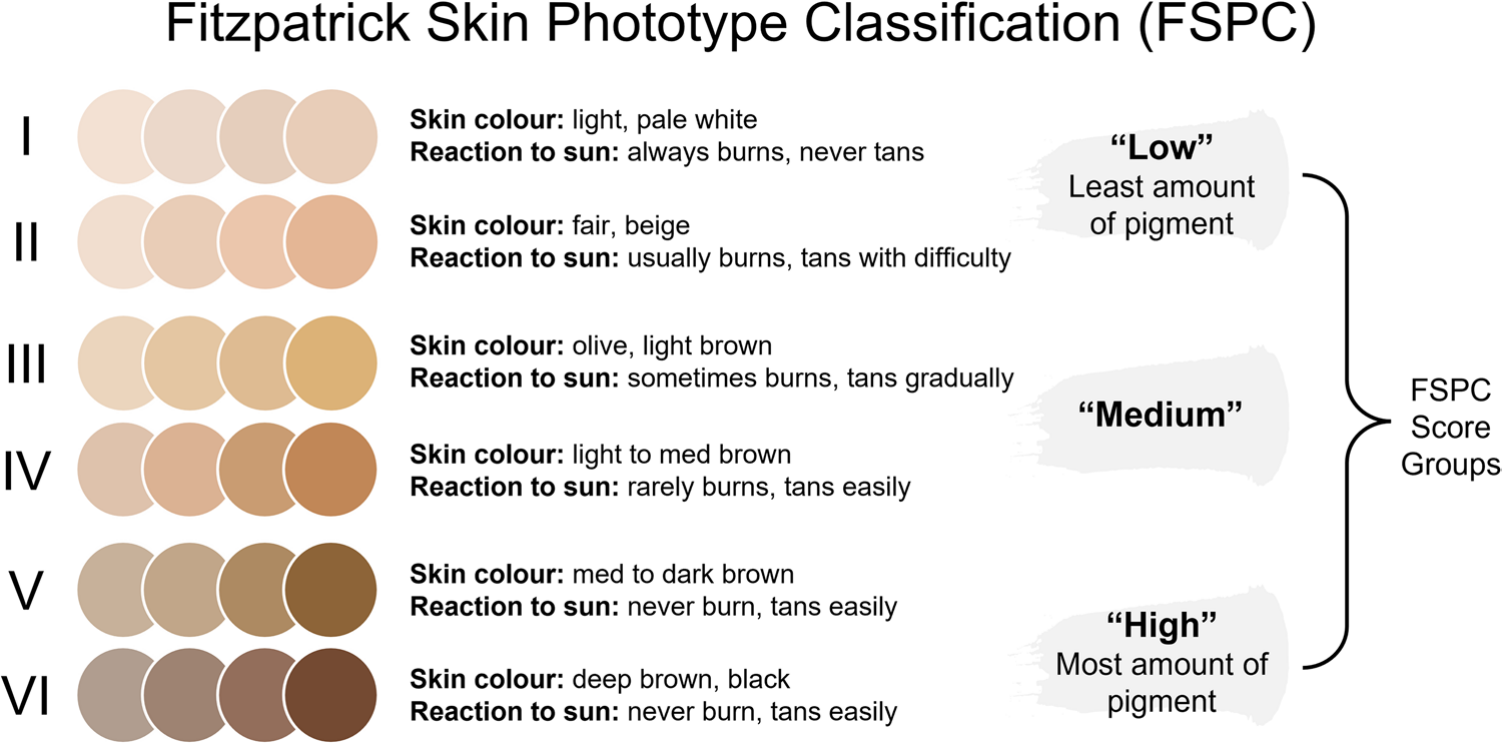
Fitzpatrick Skin Phototype Classification (FSPC) scale depicting each possible FSPC score (I–VI) and FSPC groups used in this post hoc analysis (low, medium, high). Actual skin tones corresponding to each FSPC score may vary from the examples shown here as an infinite number of skin tones exist within each category

The Fitzpatrick Skin Phototype Classification (FSPC) system classifies skin types in terms of the amount of pigment in the skin and response to sun exposure (Fig. 1) [29] and is frequently used in clinical care [17, 30]. Clinicians determined FSPC scores for each patient using their judgement and a visual color scale [22], and for this post hoc analysis, we assigned each patient to one of three FSPC groups, low (I and II), medium (III and IV), or high (V and VI) (Fig. 1).

### Reader Study: the Impact of Image Interpretation Experience

Previously, post hoc image analysis by expert readers had been performed on the 60 surgical wounds in the FLAAG trial [31]. Three “expert” readers (1 MD [surgeon], 1 DPM, 1 LPN) independently analyzed the FL images in duplicate while blinded to results of the image analysis and microbiology. The expert readers’ imaging experience included passing an advanced image interpretation test with a score >80% (score range: 83–100%) and imaging routinely for 6+ months outside the clinical trial setting (>200 wounds). The image interpretation experience of the expert readers is considered “advanced” compared to the other study clinicians because they had independently practiced FL-imaging on hundreds of wounds, while no hands-on FL-imaging practice, outside of prescribed classroom style training for this trial, was required for the “novice” readers. Two wounds were excluded for reasons previously described [31], leaving novice vs. expert image read data from 58 surgical wounds. In the current study, we evaluated these findings across FSPC groups to assess for differences in sensitivity based on image interpretation experience.

### Statistics

Sensitivity and specificity were calculated using exact Clopper-Pearson and compared (two-sided exact McNemar tests) for each FSPC group, with two-sided *p*-values based on the cumulative binomial distribution. Sensitivity and specificity were also compared between “novice” and “expert” readers for each of the FSPC groups. Post hoc comparison of sensitivity for each test between the FSPC groups was conducted using Chi-squared test, as recommended by Campbell [32] and Richardson [33]. The confidence interval (CI) was calculated according to the recommended method given by Altman et al. [34]. Categorical data were analyzed using Chi-squared tests and non-parametric continuous data was analyzed using Mann-Whitney U test or Kruskal-Wallis H test, as appropriate. False discovery rate (FDR) was conducted to correct for multiple comparisons with adjusted *p*-values (*q*-values) reported for significant findings. We set the threshold for statistical significance to *α* = 0.05 for all calculations, unless otherwise stated.

## Results

Most patients were male with a diabetic foot ulcer (DFU; 39%) or a venous leg ulcer (VLU; 30%) and the average age of the study participants was 60 years (range: 28–96 years) (Table 1). Patient skin tone varied across the entire FSPC scale (I–VI), with at least 20 patients with each possible FSPC score (Table 1).

**Table 1:** Patient demographics and wound characteristics

Demographics	Total	FSPC types I and II “low”	FSPC types III and IV “med”	FSPC types V and VI “high”
	No. (%)	No. (%)	No. (%)	No. (%)
No. patients	350	224	83	43
Mean (SD) age in years	60 (12)	61 (13)	55 (11)	59 (9)
Age range in years	28-96	28-96	28-86	37-83
Sex				
Female	125 (36)	86 (38)	21 (25)	18 (42)
Male	225 (64)	138 (62)	62 (75)	25 (58)
Wound duration				
< 3 months	106 (30)	75 (33)	20 (24)	11 (26)
3–6 months	62 (18)	36 (16)	20 (24)	6 (14)
6–12 months	56 (16)	35 (16)	16 (19)	5 (12)
12+ months	126 (36)	78 (25)	27 (33)	21 (49)
Wound type				
Diabetic foot ulcer	138 (39)	72 (32)	52 (63)	14 (33)
Venous leg ulcer	106 (30)	76 (34)	17 (20)	13 (30)
Pressure ulcer/injury	22 (6)	19 (9)	2 (2)	1 (2)
Surgical wound	60 (17)	39 (17)	10 (12)	11 (26)
Other	24 (7)	18 (8)	2 (2)	4 (9)

**Fig. 2:**
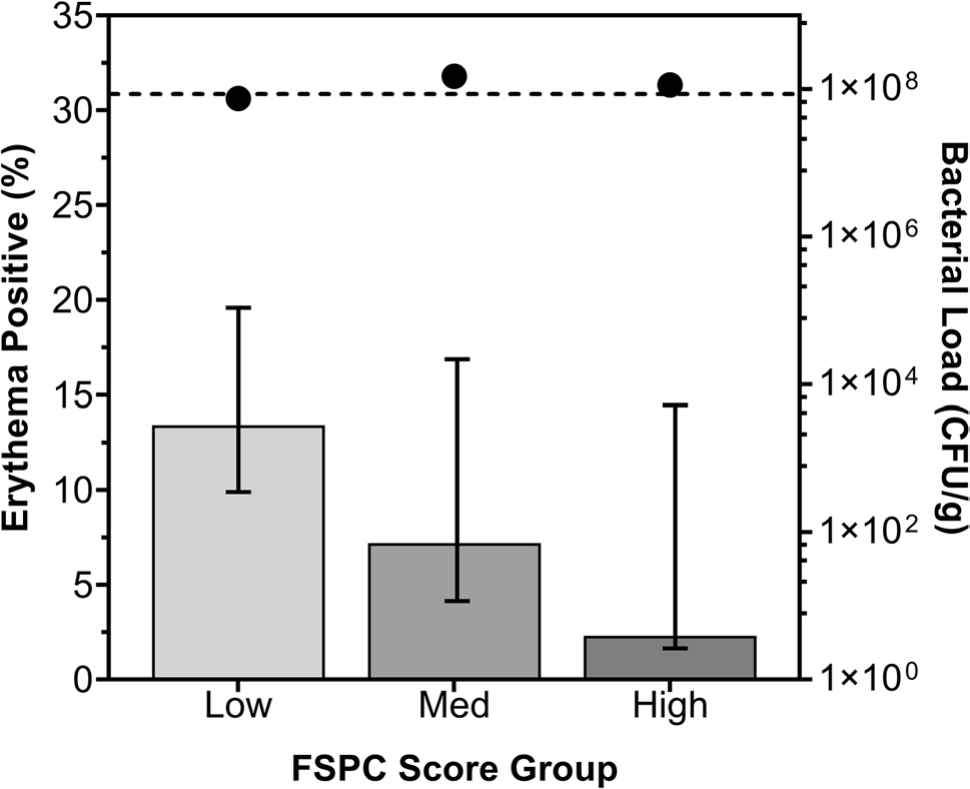
Frequency of detected erythema and average bacterial load by FSPC group. Black circles indicate median bacterial load per FSPC group; dotted line indicates average bacterial load across all study wounds (*n* = 350). Black bars represent 95% CIs

Median TBL across all study participants was 1.80 × 10^6^ CFU/g (range 0 to 7.79 × 10^9^ CFU/g). Bacterial load was not significantly different among the three FSPC groups (Kruskal-Wallis test). Notably, the frequency of erythema decreased proportionally with increasing FSPC score (Fig. 2). Despite overlapping 95% confidence intervals, Chi-squared analysis revealed a near significant difference in the frequency of erythema reporting across the FSPC groups (Table 2). A near significant difference in the incidence rate (IR) of erythema was found between the low (IR: 0.1339, [95% CI, 0.0904 to 0.1912]) and high (IR: 0.0230; [95% CI, 0.0006 to 0.1296]) FSPC groups (IRR: 0.1736) corresponding to an 87% decrease; however, the other pairwise comparisons (low vs. med, med vs. high) were not significantly different. Chi-squared analyses also revealed that the frequency of two additional CSS, wound breakdown and enlargement and delayed healing beyond expectation, varied significantly across the three FSPC groups (Table 2). Their frequencies did not follow the same trend as erythema, and did not proportionally decrease with increasing skin pigmentation; however, the high FSPC group fared the worst in both cases. There were no statistically significant differences in the frequency of all other reported CSS between FSPC groups (Table 2).

**Table 2:** Chi-squared analyses for differences in various clinical signs and symptoms (CSS) of wound infection among low (I and II), medium (III and IV), and high (V and VI) FSPC score groups

Clinical sign or symptom^ϯ^	*n*	χ^2^ statistic	*p*-value	Significance
Wound breakdown and enlargement	58	17.40	0.0002	***
low = 12.3%	28			
med = 31.3%	26			
high = 9.3%	4			
Delayed wound healing beyond expectation	185	11.86	0.0027	**
low = 50.4%	113			
med = 67.5%	56			
high = 37.2%	16			
Erythema	37	5.96	0.051	-
low = 13.4%	30			
med = 7.2%	6			
high = 2.3%	1			
Extending induration ± erythema	9	5.20	0.07	-
Bleeding, friable granulation	43	4.58	0.10	-
New or increasing pain	21	3.64	0.16	-
Increasing malodor	22	3.54	0.17	-
Wound breakdown with or without satellite lesions	19	3.56	0.17	-
Swelling	54	2.69	0.26	-
Local warmth	20	2.44	0.29	-
Epithelial bridging and pocketing in granulation tissue	12	1.77	0.41	-
Lymphangitis	3	1.70	0.43	-
Hypergranulation or excessive vascular tissue	28	1.62	0.45	-
Purulent discharge	10	1.49	0.47	-
Inflammation, swelling, or lymph glands	18	0.64	0.73	-
Malaise, lethargy, or non-specific deterioration	1	0.56	0.75	-
Crepitus	0	-	-	-
Loss of appetite	0	-	-	-

^ϯ^per International Wound Infection Institute (IWII) criteria [23]. Statistically significant at *p*<0.05 (*), *p*<0.01 (**), or *p*<0.001 (***). Number and frequency (%) per FSPC group are indicated for statistically significant (or near statistically significant at *p*<0.06) CSS

**Fig. 3:**
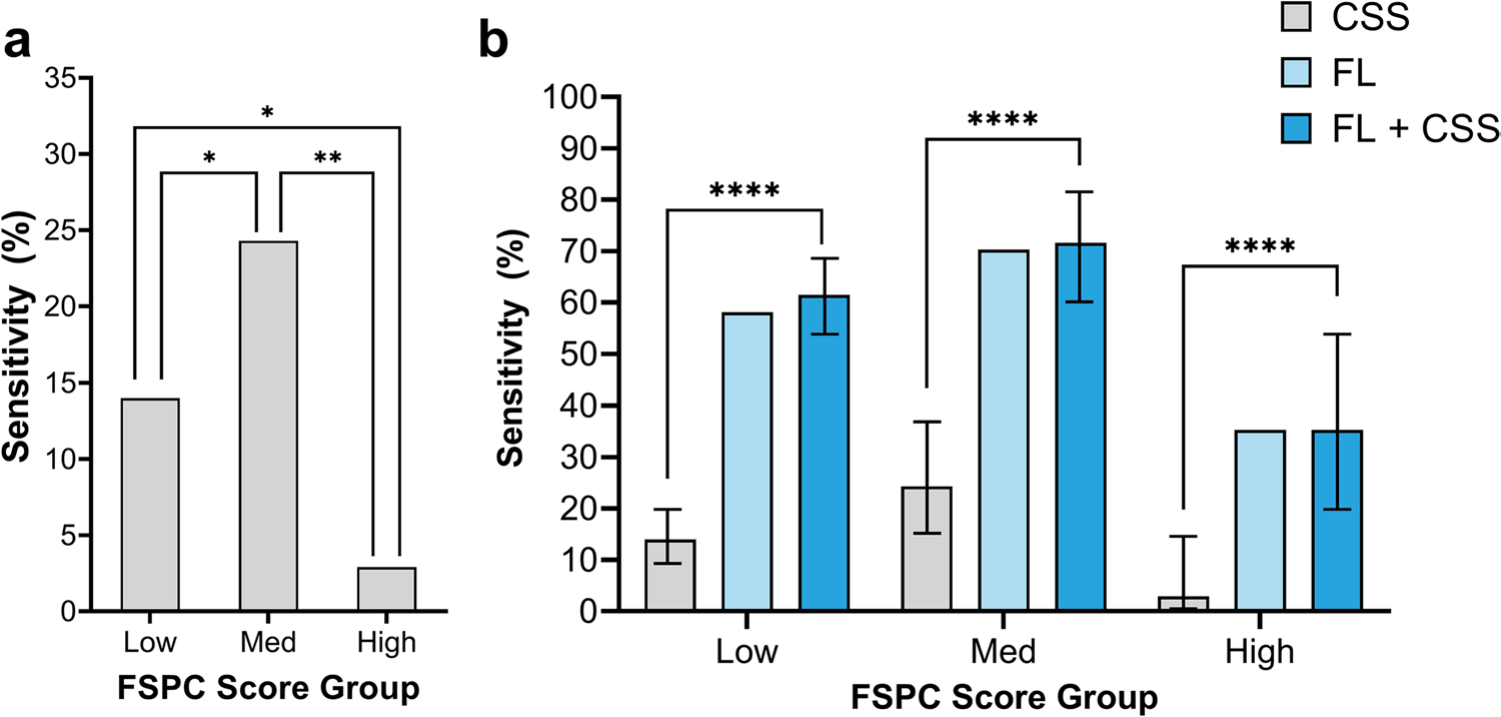
Sensitivity for detecting high bacterial loads of **a** CSS alone with inter-group statistical significance, and **b** CSS alone versus FL alone or CSS in combination with FL. CSS, infection assessment based on clinical signs and symptoms (positive if detected ≥3 CSS, as per International Wound Infection Institute guidelines [23]). FL, fluorescence imaging of bacteria >10^4^ CFU/g (positive if red and/or cyan fluorescence detected). FL+CSS, fluorescence imaging combined with clinical assessment. Low, FSPC I and II; Med, FSPC III and IV; High, FSPC V and VI. Statistically significant at *p*<0.05 (*), *p*<0.01 (**), or *p*<0.0001 (****). Black bars represent 95% CIs

**Fig. 4:**
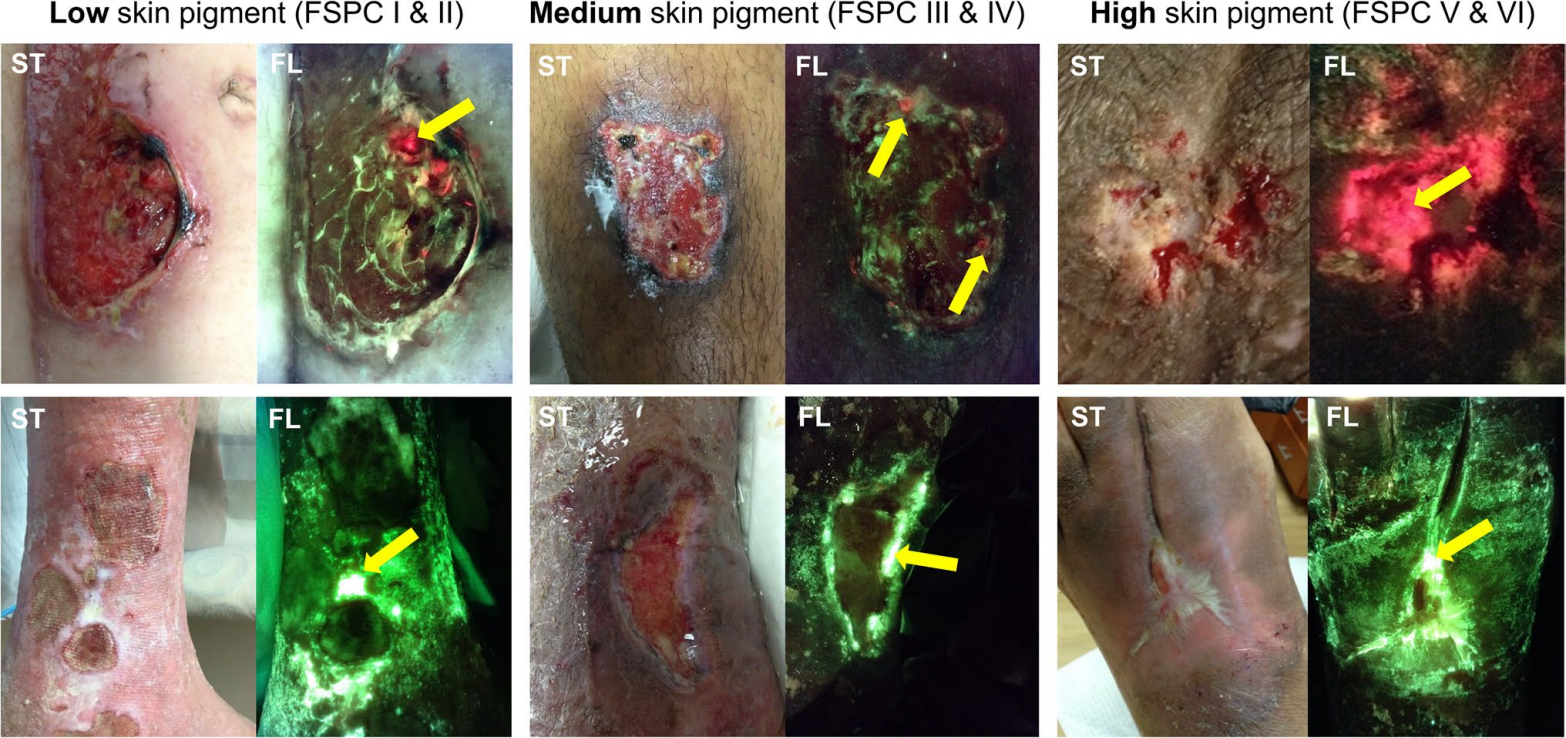
Fluorescence imaging illuminates high bacterial loads in wounds with low, medium, and high levels of skin pigment. ST, standard image; FL, fluorescence image. Yellow arrows indicate fluorescence positive regions; red signal indicates the presence of most Gram-positive and Gram-negative bacterial species at loads >10^4^ CFU/g, while cyan signal is unique to *Pseudomonas aeruginosa* [24–26]

In this study, sensitivity reflects the ability of either CSS, fluorescence imaging (FL), or CSS and FL combined (FL+CSS) to predict bacterial loads >10^4^ CFU/g (confirmed by quantitative microbiology). Using CSS assessment alone, the sensitivity to detect high bacterial loads was 14.0% in the low FSPC group, 24.3% in the medium FSPC group, and 2.9% in the high FSPC group (Fig. 3a). While there is some overlap between the 95% CIs in each group, there are statistically significant differences in these sensitivities. When FL-imaging was added to CSS assessment (FL+CSS), each of these sensitivities improved with high levels of statistical significance (Fig. 3b). This increase in sensitivity was 4.4-fold in the low FSPC group, 2.9-fold in the medium FSPC group, and peaked at 12-fold in the high FSPC group. The specificity of CSS alone was high, but not significantly different, among FSPC groups (range = 93–100%) and expanded upon incorporation of FL-imaging (range = 78–100%). It should be noted that the sensitivities of FL alone were very similar to that of FL+CSS (Fig. 3b), being identical for the high skin pigmentation group, and therefore appeared to be the primary driver of the combined sensitivity, while specificities of FL alone were reduced. FL alone was not included in the inter-group statistical comparisons as it would be inappropriate to consider imaging findings without clinical assessment context. Figure 4 provides examples of wounds which were negative for CSS but showed fluorescence indicating high bacterial loads.

Sub-analysis of the surgical wounds from this dataset (*n* = 78) was performed to compare the sensitivities of FL+CSS between “novice” and “expert” readers. Advanced image interpretation experience increased the sensitivity in the high FSPC group (*n* = 11) from zero to a clinically significant 55.6%. Sensitivity also increased significantly for the “expert” readers in the low (*n* = 37) and medium (*n* = 10) FSPC groups, from 50.0% and 57.1% up to 78.6% and 100%, respectively. Only the low FSPC group showed a statistically significant change in sensitivity, likely due to the small sample size in this sub-analysis. Specificity was not significantly different among the FSPC groups.

## Discussion

A variety of chronic wound types (138 DFUs, 106 VLUs, 60 surgical wounds, 22 PUs/PIs, and 24 other full-thickness wounds) and skin pigmentations (FSPC scores I-IV) were represented in this post hoc clinical trial analysis. Mean TBL from quantitative tissue culture did not differ between FSPC groups, and clinical assessment findings would therefore be expected to be similar across groups. However, erythema, delayed wound healing, and wound breakdown and enlargement were noted less often in patients with high FSPC scores. Detection of high bacterial loads (>10^4^ CFU/g) based on ≥3 CSS was up to 8.4-fold lower for patients with high FSPC scores (2.9%) and increased by up to 12-fold after incorporating FL-imaging, with comparably high specificity. As evidenced in the subset of surgical wounds, advanced image interpretation experience further increased this sensitivity in each FSPC group; this finding can be extrapolated to other wound types. To our knowledge, this is the first study to examine how the detection of CSS differs by skin tone.

We found that, despite equivalent wound bacterial loads, clinicians detected erythema, delayed wound healing, and wound breakdown and enlargement less frequently in patients with high skin pigmentation. Unlike erythema, diagnostic disparities in delayed wound healing and wound breakdown and enlargement cannot be directly explained by the effect of the melanin content. Unravelling the underlying causes extends beyond the scope of the present study and further research is needed to better understand those relationships. We propose that the underreporting of erythema in high FPSC patients may result from misinterpretation of the idiosyncratic presentation of erythema on diverse skin tones [4]. The word “erythema” itself incites bias. It is derived from the Greek word “erythros,” meaning red; however, capillary dilation within highly pigmented skin results in a range of hues including violet, burgundy, and deep purple [4]. It is critical for clinicians to understand how erythema, among other CSS, presents on a range of skin tones. However, clinical educational materials lack descriptors for pathological manifestations in highly pigmented skin [35, 36] and less than 5% of images in medical literature include dark skin tones [36], only slightly higher in dermatological texts (4 to 18%) [37]. This key diagnostic information is also absent from many general and wound-type specific assessment guidelines, including Infectious Diseases Society of America (IDSA) diagnostic guidelines for skin and soft tissue infections [38–42]. Only the most recently updated IWII wound infection guidelines acknowledged that skin tone can impact erythema presentation, but fails to provide images or descriptions to guide clinical examination [16]. A comprehensive review by Oozageer-Gunowa (2022) highlights the gaping lack of evidence and literature addressing racialized skin tone bias in wound care [30] and that studies in this area are exclusive to PUs/PIs [7–11], with the exception of a prospective study assessing the role of skin color on skin biomechanics in acute genital-anal injury [43]. However, even in PU/PI-specific resources, which stress that non-blanching erythema is a key indicator for early PU/PI formation [42], actionable information regarding PU/PI assessment on dark skin is lacking.

Taking into context the paucity of educational material on the appearance of infection in high skin pigmentation patients and their disproportionally poor wound outcomes, we can begin to understand the disparities in CSS assessment observed herein. The risk of infection increases with bacterial load [44, 45], and wounds in the high FSPC group were up to 8.4 times less likely to be flagged as infected (≥3 CSS) upon standard clinical inspection. Though not explored herein, the implications of unrecognized infections are staggering. Twenty percent of moderate to severely infected DFUs will proceed to amputation [46], PU/PI-associated polymicrobial bacteremia increases mortality risk by 7-fold [47], and SSIs frequently proceed to dehiscence, a severe postoperative complication which extends hospital stays and increases mortality risk [48]. Emerging literature suggests that healthcare inequities impacting people of color may be related to providers’ implicit racial/ethnic bias favoring privileged, largely White populations [49, 50]. Taken together with the aforementioned disparities in erythema, delayed healing, and wound breakdown and enlargement, we believe these results allude to a multifactorial and complex bias. This is a deep-seated issue for which there is no quick fix, but steps must be urgently taken so that the same quality of wound care is delivered irrespective of skin tone. Developing more objective and equitable wound assessment tools is a paramount step toward this much needed objective.

FL-imaging is poised to narrow this gap given its capacity to objectively detect pathogenic levels of bacteria [22, 26, 27, 51]. Owing to wavelength-specific filters in the FL-imaging device (MolecuLight), the intensity of red/cyan bacterial fluorescence is not impacted by skin tone [22]. This is a stark departure from other skin and wound assessment devices, such as those used for infrared perfusion assessment. These aim to determine the level of oxygen saturation in the vascular network, but can be prone to erroneous readings and missed diagnoses of hypoxemia on dark skin tones, as is the case with pulse oximetry [52]. Consistent with other wound imaging studies, FL-imaging revealed in our assessment pathogenic levels of bacteria otherwise missed during clinical assessment [22, 31]. FL-imaging increased detection sensitivity up to 12-fold over clinical assessment alone in the high FSPC group, and this increased even further for clinicians with advanced image interpretation experience, again with the greatest benefit to high FSPC score patients. Thus, based on the current study findings, FL-imaging appears to level the playing field when it comes to identifying at-risk, bacterial laden wounds and facilitates a more equitable approach to wound care.

Using FL-imaging to identify bacterial loads early also enables proactive care, preventing complications such as infection, and improving wound outcomes. For instance, a 2022 RCT showed that adding FL-imaging to standard clinical assessment doubled 12-week DFU healing rates [53]. Furthermore, Price et al. [54] reported increased DFU healing rates (+23%) alongside decreased systemic antibiotic prescribing (−33%), suggestive of decreased infections, and Caputo et al. report that antimicrobial prescribing decisions changed at the bedside in 41% of the visits with the use of FL-imaging [55]. Therefore, FL-imaging also has a role to play in improving outcomes across all skin types and colors.

Resulting from our findings in this study, and in addition to other guidance documents [1, 4, 17, 56], we recommend the following to improve wound infection management and care for patients across the range of skin pigmentations:

- *Inclusive wound care education*: A 2022 global survey revealed that 64% of wound-treating healthcare professionals (tissue viability nurses, podiatrists, doctors) were not aware of *any* educational materials regarding skin tone and wound care [17]. To fill this void, we recommend updated guidelines and more widespread education on the variable appearance of erythema and the other symptoms of wound infection across skin pigmentations, supported by example images and more inclusive language.
- *Equitable and objective diagnostic methods*: We recommend adoption of FL-imaging to detect bacterial loads that delay wound healing and increase the risk of complications such as infection, sepsis, amputation, morbidity, and mortality [57–60]. Previous studies have definitively shown that CSS fail to alert to high bacterial loads [22, 61]; we show here that this failure is most pronounced in patients with the highest levels of skin pigment. This disparity is a likely contributor to the higher amputation rates and overall poorer wound outcomes in the Black patient population [7, 8, 10, 11]. At present, FL-imaging is the only technology evidenced to ameliorate this racial inequity in wound assessment of infection, at least in part.
- *Higher suspicion of infection in wound patients with high skin pigmentation*: Echoing the first of Black and Simende’s tips for assessing highly pigmented skin [56], we recommend a high index of suspicion when assessing dark skin, understanding that indicators of infection may be variable and subtle. It is critical to rely on other senses, especially touch (e.g., feeling for warmth or swelling), particularly in the absence of overt visual cues [4].

This study was strengthened by a large sample size (*n* = 350), with at least 20 patients with each of the six possible FSPC scores. As we included a range of chronic wound types assessed by 20 clinicians with minimal exclusion criteria, these results directly represent the real-world chronic wound patient population. To our knowledge, this is the largest study to examine the impact of skin tone on erythema presentation in chronic wounds and the first to include chronic wounds beyond PUs/PIs. A limitation is that we used a bacterial threshold (10^4^ CFU/g) to inform on infection presentation and risk; however, there is no microbiological threshold for diagnosing infection in chronic wounds and clinical symptoms that would indicate infection frequently fail to mount in the largely immunocompromised wound care patient population [22, 61, 62]. As this was a post hoc analysis of a completed single-timepoint clinical trial, we could not assess wound outcomes and are therefore unable to report the impacts of missed high bacterial loads in those patients with high FSPC scores. Finally, the reliability of the widely used FSPC system has been under scrutiny [63]; however, this clinical trial was designed together with the FDA and the FSPC scale was the classification system the FDA recommended at study conception.

## Conclusions

Clinicians were less likely to flag bacterial-laden wounds on highly pigmented skin as being problematic—delaying treatment and, potentially, worsening wound outcomes and increasing morbidity and mortality risk. These findings demonstrate the healthcare inequities facing racial/ethnic minority patient populations, perpetuated in part by inaccurate or incomplete clinical resources and terminology, as well as providers’ implicit racial bias. We present recommendations to facilitate equitable and improved wound-infection management, such as FL-imaging to enhance bacterial load detection across all skin tones. More research is needed to understand how the lack of knowledge on clinical assessment of individuals with highly pigmented skin impact rates of diagnosis and outcomes in wound care and in other fields of medicine.

## Data Availability

To protect patient confidentiality, the clinical trial data are not publicly available; however**,** they are available from the authors upon reasonable request and with permission of MolecuLight, Inc.
